# Modeling the critical care demand and antibiotics resources needed during the Fall 2009 wave of influenza A(H1N1) pandemic

**DOI:** 10.1371/currents.RRN1133

**Published:** 2009-12-07

**Authors:** Duygu Balcan, Vittoria Colizza, Andrew C. Singer, Christos Chouaid, Hao Hu, Bruno Gonçalves, Paolo Bajardi, Chiara Poletto, Jose J. Ramasco, Nicola Perra, Michele Tizzoni, Daniela Paolotti, Wouter Van den Broeck, Alainjacques Valleron, Alessandro Vespignani

**Affiliations:** ^*^Indiana University, Bloomington, IN; ^†^Computational Epidemiology Lab, ISI Foundation, Turin, Italy; ^‡^Centre for Ecology & Hydrology, UK; ^§^Department of Physics and Center for Complex Networks and Systems Research, Indiana University; ^¶^Center for Complex Networks Systems Research; ^#^Computational Epidemiology Laboratory, Institute for Scientific Interchange; ^**^Institute for Scientific Interchange Foundation, Turin; ^††^Complex Networks Lagrange Laboratory and Computational Epidemiology Laboratory, ISI Foundation, Turin, Italy; ^‡‡^Complex Networks and Systems Research School of Informatics and Computing; ^§§^Computational Epidemiology Laboratory, ISI Foundation, Turin, Italy; ^¶¶^Institute for Scientific Interchange - ISI; ^##^ISI Foundation; Universite Pierre et Marie Curie and Indiana University

## Abstract

While the H1N1 pandemic is reaching high levels of influenza activity in the Northern Hemisphere, the attention focuses on the ability of national health systems to respond to the expected massive influx of additional patients. Given the limited capacity of health care providers and hospitals and the limited supplies of antibiotics, it is important to predict the potential demand on critical care to assist planning for the management of resources and plan for additional stockpiling. We develop a disease model that considers the development of influenza-associated complications and incorporate it into a global epidemic model to assess the expected surge in critical care demands due to viral and bacterial pneumonia. Based on the most recent estimates of complication rates, we predict the expected peak number of intensive care unit beds and the stockpile of antibiotic courses needed for the current pandemic wave. The effects of dynamic vaccination campaigns, and of variations of the relative proportion of bacterial co-infection in complications and different length of staying in the intensive care unit are explored.

## Introduction

Official national reports from several countries in the Northern Hemisphere signal increasing influenza activity, as measured by the rise in the number of cases and patients requiring medical attention [1] [2] [3]. From the pandemic wave in the Southern Hemisphere [4] and the current activity [1] [2] [3], a clearer picture of the severity of the disease has emerged in different geographic zones. Hospitalizations and number of cases requiring admission to intensive care unit (ICU) have been recorded, generating a picture of disease progression and illness severity requiring medical attention, hospitalization or critical care [4] [5] [6] [7] [8]. This data is also crucial for clinically assessing influenza-associated complications to update patient management recommendations [9] [10] [11] [12] [13] [14] [15].

 Given the limited availability of critical care facilities and medical resources, it is important to assess the expected potential burden on health services in order to face possible emergencies requiring highly specialized personnel and care units, for usually long and costly stays [16]. While a lot of work has been conducted on stockpiling and planning for deployment and distribution of antiviral drugs in case of an emerging influenza pandemic [17] [18] [19] [20] [21] [22] [23] [24] [25], much less attention has been devoted to the role of bacterial pneumonia in pandemic planning, particularly in terms of stockpiling antimicrobial drugs [26] [27]. Antibiotics are generally available through short supply chains able to fulfill average just-in-time requests. The pandemic wave is however expected to lead to a large increase in the usage pattern of antibiotics possibly relevant in the management of stockpiles not only during the peak phase but also during the decreasing trend of the epidemic activity. Based on the available knowledge of the severity of the disease and its associated complications, we present a computational study that explicitly considers the development of complications in order to estimate the predicted request of ICU resources and antibiotics needed to treat complications in several countries of the Northern Hemisphere, during the current Fall 2009 pandemic wave. 

## Methods

### Baseline model

We use the global epidemic and mobility structured metapopulation model (GLEaM) [28] [29] to provide pandemic scenarios and quantify the expected demand for critical care resources. The model is based on a meta-population approach [17] [30] [31] [32] [33] [34] [35] [36] [37] [38] [39] in which the world is divided into geographical regions defining a subpopulation network where connections among subpopulations represent the individual fluxes due to the transportation and mobility infrastructure. GLEaM integrates three different data layers [28] [29]: (i) the population layer that integrates census areas for a total of 3362 subpopulations in 220 countries of the world; (ii) the human mobility layer that integrates both commuting flows collected from various sources in more than 30 countries and the airline traffic provided by IATA and OAG [28] [29]; (iii) the disease dynamics layer. 

The model simulates short range mobility between subpopulations with a time scale separation approach that defines the effective force of infections in connected subpopulations [17] [29] [40] [41]. The airline  mobility from one subpopulation to another is modeled by an individual based stochastic procedure in which the number of passengers of each compartment traveling from a subpopulation j to a subpopulation l is an integer random variable defined by the actual data from the airline transportation database [17]. The infection dynamics takes place within each subpopulation. We adopt a *SEIR*-like model [42] in which we include vaccinated individuals and specific compartments for influenza associated complications. We also consider separate compartments for symptomatic traveling and not traveling, as well as asymptomatic individuals in each subpopulation. All transitions are modeled through binomial and multinomial processes to ensure the discrete and stochastic nature of the processes [17] [29]. Asymptomatic individuals are considered as a fraction \begin{equation*}p_{a}= 33\%\end{equation*} of the   infectious individuals generated in the model and assumed to infect with a relative infectiousness of \begin{equation*}r_{\beta } = 50\%\end{equation*} [19] [43]. Change in traveling behavior after the onset of symptoms is modeled with the probability \begin{equation*}1-p_{t}\end{equation*} set to 50% that individuals would stop travelling when ill [19] (see Figure 1 for a detailed description of the compartmentalization). Initial conditions are defined by setting the start of the epidemic in La Gloria in Mexico on 18 February 2009 [29] [43] [44]. In the model we use values of generation time interval and transmissibility according to the estimates of [29] [45]. In particular, we use the reproductive number* R_0_=1.75* with the generation interval set to 3.6 days (average latency period of 1.1 days and an average infectious period of 2.5 days). Those values are obtained by using the model to perform maximum likelihood analysis of the parameters against the actual chronology of newly infected countries as detailed in Ref. [29]. The method is computationally intensive as it involves a Monte Carlo generation of the distribution of arrival time of the infection in each country based on the analysis of 1 Million worldwide simulations of the pandemic evolution with the GLEaM model. It is important to remark that the best estimate of the reproductive number refers to the reference value that has to be rescaled by the seasonality scaling function. Seasonality is considered in the model by means of a sinusoidal forcing of the reproductive number, with a scaling factor ranging from α_min_ during Summer season to α_max_ during Winter season [33]. Here we consider α_max_= 1.1 and α_min_ in the range 0.6 to 0.7, that is the best estimate obtained from the correlation analysis on the chronology of 93 countries seeded before June 18 in Ref. [29]. This seasonal scaling provides an effective reproductive number in the Northern hemisphere in the range 1.2 to 1.6 in the spring/fall months, in agreement with published estimates of the reproductive number. The best estimates of the model parameters provide predictions for the influenza activity peak in countries in the Northern Hemisphere in October/November in the baseline scenario [29], consistent with the influenza activity now being observed in surveillance reports [1] [2] [3]. In the following we will use the reference value α_min_=0.6. A full discussion of the model’s limitations and of the sensitivity analysis of the model’s assumptions is reported in Ref. [29].

### Vaccination

 We model the administration of vaccines through a dynamic vaccination campaign with a uniform daily rate r_v _of distribution to the population in countries where doses are available, till their exhaustion. We explore two values for the daily distributions rate, r_v_=0.1% consistent with the current availability of doses and distribution in several countries, and r_v_=1% based on the distribution policies planned during Summer 2009 on the timing of vaccine development and testing [46]. We assume the administration of a single dose of vaccine, providing protection with a delay of 2 weeks. A full description of the vaccination implementation and sensitivity analysis is reported in Ref. [46].

### Influenza-associated complications

 Following the most recent estimates of the severity of H1N1 pandemic, we assume a complication rate of 15% of clinical cases [7], a hospitalization rate of 0.5% of clinical cases [6], and an ICU admission rate of 15% of hospitalized patients [3]. We model influenza-related pneumonia as a complication associated to influenza infection, considering two main types of pneumonia – primary viral pneumonia and secondary bacterial pneumonia. While bacterial coinfection was shown to be the predominant cause of death in previous influenza pandemics [47], its presence in the severe cases analyzed since the start of the outbreak range from almost no evidence in the early reviews [9] [10] [11], to about 10% [13], 33% or larger proportions [14] [15] of the cases presenting influenza-associated complications. These fluctuations in the role of bacterial pneumonia might be due to the difficulty of testing for specific bacterial diagnosis, or to the use of antibiotics prior to routine clinical tests. Given the uncertainty on the cause of pneumonia at this stage of the epidemic evolution, we assume a proportion of bacterial pneumonia in cases showing complications in the range of α= 33-50%, with a sensitivity exploring a 10% proportion. Under pandemic conditions, it is assumed that very small differences will be implemented in the management and treatment of the patients with either types of pneumonia, as the diagnosis of influenza-associated complications will be mostly based on clinical findings and most prescribing will be empirical, based on both antibacterial therapy and antiviral medications [48]. Multiple subsequent stages of pneumonia course are modeled according to the CURB-65 classification score [49] as reported in Table 1, and different progressions are assumed to take into account both viral and bacterial pneumonia (see Figure 1). It is also worth remarking that the model does not consider social structure in the subpopulations, therefore the effect of prioritized distribution of  vaccines to individuals belonging to  risk groups in reducing the number of hospitalizations and deaths is not considered in the present study. These assumptions represent a necessary trade-off for the computational efficiency of the model that allows to perform parameter estimations fitting the worldwide pattern of the pandemic [29], explore several scenarios under different conditions, and perform sensitivity analysis on the assumptions. Once the disease parameters and initial conditions are defined, GLEaM generates in-silico epidemics for which we can gather information such as incidence and prevalence of all stages considered in the compartmentalization, for each subpopulation in the world and with a time resolution of one day. All results shown in the following sections are obtained from the statistics based on at least 2,000 stochastic runs of the model.

## Results and Discussion

 Based on the available knowledge of complication, hospitalization and ICU rates, and the relative proportion of bacterial vs. viral pneumonia, the simulation results allow the measure of the predicted need of beds in intensive care units, and provide estimates of the corresponding courses of antibiotics needed. Figure 2 shows the time evolution of the predicted prevalence of ICU occupancy for a given set of countries. In the baseline case, when no intervention is implemented, the ICU prevalence peak ranges between approximately 5 and 7 ICU beds per 100,000 people. These values are well below the national average capacity of some countries, such as e.g. the United States with a total of about 20 ICU beds per 100,000 [50] and Germany with an average of approximately 28 ICU beds per 100,000 [51]. The predicted need is slightly lowered if a 0.1% dynamic vaccination is considered, and would be reduced to values in the range of 3.6 to 4.8 ICU beds per 100,000 if we assume r_v_=1%, below the national average number of ICU beds of many European countries [16]. While the predicted ICU beds needs are averaged at the country level to conform with the capacity data, it is however important to note that the impact and the potential occurrence of critical situations strongly depends on the geographic distribution of the critical care resources, with areas that might have access to a larger number of intensive care units than others (see for example Ref. [52]). Moreover, a direct comparison between the simulated demand and critical care availability is made difficult by the lack of a standard definition for intensive care unit beds, and the large variations observed in both numbers of beds and volume of admission between countries in North America and Western Europe [16].

 The results shown in Figure 2 are based on an average ICU length of staying equal to L_ICU_=7 days. Since there is a large variation in this parameter, with cohort studies showing median duration of 7 days and interquartile range up to approximately 2 weeks [15], we also explored the effect of considering longer lengths of staying, L_ICU_=10 and L_ICU_=14 days. The longer bed occupancy would inevitably lead to an increase in the need of ICU beds at peak, in the range of approximately 9 to 12 per 100,000 persons in the case of 14 days of average ICU duration (see Table 2). 

 Table 3 reports the number of antibiotics courses needed daily at the peak of the requests, and the total size predicted to be used at the end of the pandemic wave, based on the empirical guidelines of the British Thoracic Society [49] [53] and broken down by the stage of severity of pneumonia. A single course of antibiotics is defined as the combination of antimicrobial drugs considered in the treatment regimen for the suggested duration (see Table 1). In the case of non severe pneumonia, the predicted need for antibiotics at peak usage is in the range of [150-230] courses per 100,000 with variations depending on the country under study, under the assumption that no intervention is considered. The total size of antibiotics courses predicted to be used in the current Fall 2009 pandemic is in the range of [6,337-7,149] per 100,000, which needs to be compared with the available stockpiles of antibiotics courses to cover high-risk groups. Many countries however do not possess nation-wide antibiotic supplies, and the estimates contained in Table 3 can therefore be considered as guidelines to assess the expected needs during the remaining evolution of the pandemic wave with respect to the present usage pattern and available resources.

 Along with anecdotal reports indicating ICUs being overwhelmed by the sudden surge of H1N1 cases with severe complications [54], studies on the Winter experience in the Southern Hemisphere during the H1N1 pandemic wave confirm a substantial impact on ICUs, with the maximum number of ICU beds occupied by region in Australia and New Zealand ranging between 0.63 and 1.1 per 100,000 inhabitants [15]. These values are smaller than the ICU demands predicted for the Fall wave in the Northern Hemisphere. It is important to note, however, that the used model does not take into account the population structure (age dependent attack rates), risk groups and prior immunity thus likely overestimating the global attack rate of the pandemic. Furthermore we do not include in the model mitigation factors (e.g. social distancing, targeted school closures, etc.) that might have contributed to the reduction of the overall burden on the critical care facilities in the Southern Hemisphere; a similar reduction on burden could also be seen in the Northern Hemisphere.

 Accurate descriptions of expected scenarios are important to define and quantify the expected increase in the needs for healthcare infrastructure and medical resources. With the uncertainties on the evolution of the current pandemic wave decreasing, these estimates can be used to better plan for potential additional resources that might be needed in a short time, both at the peak time and after the peak activity has been reached. A full comparison and understanding of similarities and differences of the Winter pandemic waves in the two Hemispheres will then be crucial for understanding the impact of H1N1 pandemic on the population and on the health care infrastructure in different settings.

## Competing interests

 AV is consulting and has a research agreement with Abbott for the modeling of H1N1 diffusion. The other authors have declared that no competing interests exist.

## Authors’ contributions

 DB, ACS, CC, A-JV contributed to conceiving and designing the study, and helped to draft the manuscript. DB performed numerical simulations and statistical analysis, and contributed to the data integration. HH, BG, PB, CP, JJR, DP, NP, MT, WVdB contributed to data tracking and integration, and to the estimation of disease parameters. AV and VC conceived, designed and coordinated the study, contributed to the analysis and methods development and drafted the manuscript. All authors read and approved the final manuscript.

## Correspondence

Correspondence to vcolizza@isi.it or alexv@indiana.edu

## Acknowledgments

 The authors thank IATA and OAG for providing their databases. 

## Funding

 This work has been partially funded by the NIH R21-DA024259 award, the Lilly Endowment grant 2008 1639-000, and the DTRA-1-0910039 award to AV; the EC-ICT contract no. 231807 (EPIWORK), and the EC-FET contract no. 233847 (DYNANETS)  to AV, VC; the ERC Ideas contract n.ERC-2007-Stg204863 (EPIFOR) to VC; the Natural Environment Research Council – Knowledge Transfer Initiative (PREPARE) contract no. NE/F009216/1 to ACS.

## Figures

**Figure:**
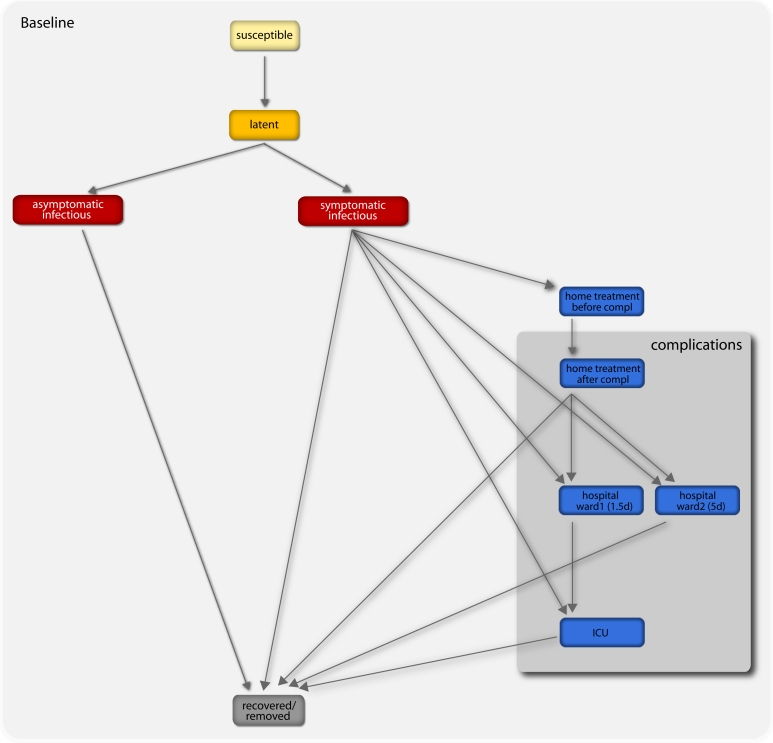
** Figure 1: Diagram flow of the transmission model.** A susceptible individual interacting with an infectious person may contract the illness and enter the latent compartment where he is infected but not yet infectious. At the end of the latency period, each latent individual becomes infectious entering the symptomatic compartment with probability (1-p_a_) or becoming asymptomatic with probability p_a_. Asymptomatic individuals infect with a reduced transmission rate. A fraction (1-p_t_) of the symptomatic individuals would stop traveling when ill. A full description of the parameter values is reported in Ref. [29]. If vaccines are available, a fraction equal to r_v_ of the susceptible population enters the susceptible vaccinated compartment each day. A similar progression to the baseline compartmentalization is considered if infection occurs (see Ref. [46]). The model assumes that infectious individuals might develop complications with a rapid progression to severe conditions requiring hospitalization or ICU admission (i.e. second and third stage of the complications tree, respectively), or home treatment (i.e. first stage) with pneumonia symptoms appearing during the early convalescent period of the influenza infection [49]. The compartments 'hospital ward 1' and 'hospital ward 2' refer to different lengths of staying of the patient in the hospital ward (see Table 1), depending on subsequent worsening of symptoms or direct recovery, respectively. Progressions from one stage to the others is modeled according to the average length of staying in each compartment as obtained from clinical studies [13] [15] (see also Table 1) and based on the available estimates of complication, hospitalization and ICU admission rates [3] [6] [7].

**Figure:**
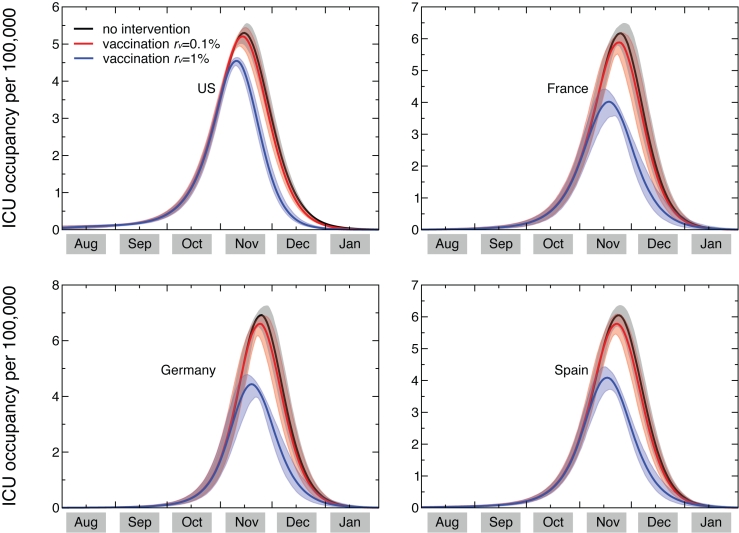
** Figure 2: Time evolution of the ICU occupancy in a set of countries**. ICU occupancy measures the predicted need of ICU beds per 100,000 persons. Results for the United States, France, Germany, and Spain are shown. The three profiles per each country refer to the predicted ICU occupancy in the baseline case when no intervention is implemented, and in case dynamic vaccination campaigns with distribution rates r_v_=0.1% and r_v_=1% are considered. Solid curves correspond to the median profiles and the shaded areas to the 95% reference range obtained from 2,000 stochastic simulations. The average ICU length of staying is assumed equal to 7 days [15].

## Tables

**Table:** 

**Severity of complications**	**Assessment**	**Recommended** **action /** **compartmentalization **	**Average duration**
non-severe pneumonia	CURB-65=0-2	home treatment or supervised outpatient treatment	3.5 days [9]
severe pneumonia	CURB-65=3 or presence of bilateral lung infiltrates on chest x ray	hospital ward	1.5 days to ICU admission (hospital ward 1), 5 days to recovery (hospital ward 2) [13]
	CURB-65=4-5 or bilateral chest x ray changes	ICU	7, 10, 14 days [13] [15]

**Table 1: Severity assessment, recommended action, and estimated durations assumed in the model. **We refer to CURB-65 score as the method used to determine the management of influenza-related complications in patients admitted to hospital [49]. CURB-65 score is calculated by assigning one point for each of the following: Confusion (mental test score of ≤8, or new disorientation in person, place or time),Urea >7 mmol/l, Respiratory rate ≥30/min, Blood pressure (SBP<90 mmHg or DBP≤60 mmHg), Age ≥65 years. Three subsequent stages are defined to model complications, based on the recommended action. Patients with bilateral lung infiltrates on chest radiography consistent with viral pneumonia are assumed to be managed as severe pneumonia, regardless of CURB-65 score [49]. The preferred empirical antibiotic regimens for treatment of patients in each stage are based on the guidelines issued by the British Thoracic Society [49] [53]. Patients in home treatment and hospital ward are assumed to take co-amoxiclav 625mg tds PO or doxycycline 200mg stat and 100mg od PO for 7 days, and patients in ICU are assumed to take co-amoxiclav 1.2g tds IV or cefuroxime 1.5g tds IV or cefotaxime 1g tds IV plus Macrolide (erythromycin 500mg qds IV or clarithromycin 500mg bd IV) for 10 days. All patients at all stages of severity of complications are also expected to receive antivirals, with a dosage of 2 tablets per day. 

**Table:** 

**ICU occupancy at peak (per 100,000)**									
**Country**	**Baseline**			**Vaccination campaigns**					
** **	** **			**0.1%**			**1%**		
	**7 days**	**10 days**	**14 days**	**7 days**	**10 days**	**14 days**	**7 days**	**10 days**	**14 days**
US	[5.0-5.6]	[6.8-7.5]	[8.7-9.7]	[5.0-5.5]	[6.7-7.3]	[8.6-9.4]	[4.5-4.6]	[5.9-6.2]	[7.6-7.9]
UK	[5.7-6.5]	[7.6-8.6]	[9.9-11.0]	[5.5-6.2]	[7.4-8.2]	[9.6-10.5]	[3.9-4.6]	[5.2-6.1]	[6.7-7.7]
Canada	[5.0-5.7]	[6.7-7.6]	[8.7-9.9]	[4.8-5.5]	[6.5-7.3]	[8.5-9.5]	[3.8-4.4]	[5.1-5.8]	[6.5-7.3]
France	[5.9-6.6]	[7.9-8.7]	[10.2-11.2]	[5.7-6.2]	[7.6-8.3]	[9.8-10.6]	[3.6-4.4]	[4.9-5.9]	[6.3-7.4]
Italy	[6.5-7.1]	[8.6-9.4]	[11.0-12.0]	[6.2-6.7]	[8.2-8.9]	[10.5-11.3]	[3.6-4.5]	[4.8-5.9]	[6.1-7.4]
Spain	[5.8-6.4]	[7.8-8.6]	[10.0-11.0]	[5.6-6.1]	[7.5-8.2]	[9.6-10.5]	[3.8-4.5]	[5.1-5.9]	[6.5-7.5]
Germany	[6.6-7.3]	[8.8-9.7]	[11.2-12.2]	[6.4-7.0]	[8.5-9.2]	[10.8-11.6]	[4.0-4.8]	[5.4-6.4]	[6.8-8.0]

**Table 2: Predicted need of ICU beds in the baseline case scenario and in the case of vaccination campaigns. **The 95% reference range (RR) of the daily number of occupied ICU beds per 100,000 is reported at its peak for several countries in the Northern Hemisphere.****

**Table:** 

**Antibiotic usage – baseline**						
**Country**	**Daily administered AB courses at peak (per 100,000)**			**Total administered AB courses at the end of pandemic wave (per 100,000)**		
	**Pneumonia stage I**	**Pneumonia stage II**	**Pneumonia stage III**	**Pneumonia stage I**	**Pneumonia stage II**	**Pneumonia stage III**
US	[152-171]	[4.4-4.9]	[0.8-0.9]	[6,196-6,455]	[183-191]	[31.7-33.0]
UK	[176-197]	[5.1-5.8]	[0.9-1.1]	[6,529-6,845]	[193-203]	[33.3-35.1]
Canada	[150-170]	[4.4-5.0]	[0.8-1.0]	[6,508-6,755]	[192-200]	[33.0-34.8]
France	[184-201]	[5.3-5.9]	[1.0-1.1]	[6,611-6,906]	[195-204]	[33.7-35.4]
Italy	[202-221]	[5.8-6.4]	[1.1-1.2]	[6,758-6,981]	[200-206]	[34.4-35.8]
Spain	[178-195]	[5.2-5.7]	[0.9-1.1]	[6,584-6,815]	[194-202]	[33.4-35.1]
Germany	[208-230]	[5.9-6.6]	[1.1-1.2]	[6,739-6,990]	[199-207]	[34.4-35.8]
**Antibiotic usage – vaccination with r_v_** **=0.1%**						
**Country**	**Daily administered AB courses at peak (per 100,000)**			**Total administered AB courses at the end of pandemic wave (per 100,000)**		
	**Pneumonia stage I**	**Pneumonia stage II**	**Pneumonia stage III**	**Pneumonia stage I**	**Pneumonia stage II**	**Pneumonia stage III**
US	[151-166]	[4.4-4.8]	[0.8-0.9]	[6,005-6,220]	[177-184]	[30.7-31.9]
UK	[170-186]	[4.9-5.4]	[0.9-1.0]	[6,297-6,540]	[186-193]	[32.1-33.6]
Canada	[147-164]	[4.3-4.9]	[0.8-0.9]	[6,278-6,457]	[185-191]	[31.8-33.3]
France	[176-188]	[5.1-5.5]	[0.9-1.0]	[6,357-6,585]	[188-195]	[32.3-33.8]
Italy	[191-206]	[5.5-6.0]	[1.0-1.1]	[6,481-6,633]	[191-196]	[32.9-34.1]
Spain	[171-185]	[5.0-5.4]	[0.9-1.0]	[6,335-6,511]	[187-193]	[32.1-33.6]
Germany	[200-216]	[5.7-6.2]	[1.0-1.2]	[6,476-6,654]	[191-197]	[33.0-34.2]
**Antibiotic usage – vaccination with r_v_** **=1%**						
**Country**	**Daily administered AB courses at peak (per 100,000)**			**Total administered AB courses at the end of pandemic (per 100,000)**		
	**Pneumonia stages I**	**Pneumonia stage II**	**Pneumonia stage III**	**Pneumonia stages I**	**Pneumonia stage II**	**Pneumonia stage III**
US	[140-144]	[4.0-4.1]	[0.7-0.8]	[4,801-4,862]	[142-144]	[24.5-25.0]
UK	[120-140]	[3.5-4.1]	[0.6-0.8]	[4,452-4,762]	[131-141]	[22.7-24.5]
Canada	[121-133]	[3.5-3.9]	[0.6-0.8]	[4,517-4,732]	[133-140]	[22.9-24.4]
France	[110-136]	[3.2-4.0]	[0.6-0.7]	[4,390-4,682]	[130-139]	[22.4-24.0]
Italy	[110-136]	[3.2-4.0]	[0.6-0.7]	[4,230-4,539]	[125-134]	[21.5-23.3]
Spain	[116-137]	[3.4-4.0]	[0.6-0.8]	[4,429-4,652]	[131-137]	[22.5-24.0]
Germany	[126-150]	[3.6-4.3]	[0.7-0.8]	[4,311-4,655]	[127-138]	[22.0-23.9]

**Table 3: Predicted usage pattern of antibiotics in the baseline case scenario and in the case of vaccination campaigns. **The 95% RR of the daily number of administered antibiotics courses per 100,000 at its peak is reported, along with the total amount predicted to be administered by the end of the pandemic wave. Results are shown for several countries in the Northern Hemisphere, broken down for different stages of influenza-associated complications. Pneumonia stages I, II and III corresponds to home-treatment (or supervised outpatient treatment), hospital wards and ICU, respectively (see Figure 1 and Table 1).
